# Complications and Comorbidities of Acromegaly—Retrospective Study in Polish Center

**DOI:** 10.3389/fendo.2021.642131

**Published:** 2021-03-16

**Authors:** Małgorzata Rolla, Aleksandra Jawiarczyk-Przybyłowska, Jowita Halupczok-Żyła, Marcin Kałużny, Bogumil M. Konopka, Izabela Błoniecka, Grzegorz Zieliński, Marek Bolanowski

**Affiliations:** ^1^ Department of Endocrinology, Diabetes and Isotope Therapy, Wroclaw Medical University, Wrocław, Poland; ^2^ Department of Biomedical Engineering, Faculty of Fundamental Problems of Technology, Wroclaw University of Science and Technology, Wrocław, Poland; ^3^ Department of Endocrinology, Diabetes and Isotope Therapy, University Clinical Hospital, Wrocław, Poland; ^4^ Department of Neurosurgery, Military Institute of Medicine, Warsaw, Poland

**Keywords:** acromegaly, complication, comorbidity, pituitary adenoma, IGF-I, GH

## Abstract

**Introduction:**

In acromegaly, chronic exposure to impaired GH and IGF-I levels leads to the development of typical acromegaly symptoms, and multiple systemic complications as cardiovascular, metabolic, respiratory, endocrine, and bone disorders. Acromegaly comorbidities contribute to decreased life quality and premature mortality. The aim of our study was to assess the frequency of acromegaly complications and to evaluate diagnostic methods performed toward recognition of them.

**Materials and Methods:**

It was a retrospective study and we analyzed data of 179 patients hospitalized in the Department of Endocrinology, Diabetes and Isotope Therapy in Wroclaw Medical University (Poland) in 1976 to 2018 to create a database for statistical analysis.

**Results:**

The study group comprised of 119 women (66%) and 60 men (34%). The median age of acromegaly diagnosis was 50.5 years old for women (age range 20–78) and 46 for men (range 24–76). Metabolic disorders (hyperlipidemia, diabetes, and prediabetes) were the most frequently diagnosed complications in our study, followed by cardiovascular diseases and endocrine disorders (goiter, pituitary insufficiency, osteoporosis). BP measurement, ECG, lipid profile, fasting glucose or OGTT were performed the most often, while colonoscopy and echocardiogram were the least frequent.

**Conclusions:**

In our population we observed female predominance. We revealed a decrease in the number of patients with active acromegaly and an increase in the number of well-controlled patients. More than 50% of patients demonstrated a coexistence of cardiac, metabolic and endocrine disturbances and only 5% of patients did not suffer from any disease from those main groups.

## Introduction

Acromegaly is a rare endocrine disease associated with elevated growth hormone (GH) and insulin-like factor I (IGF-I) levels, mainly due to a pituitary adenoma (1). Hypersecretion of GH and IGF-I leads to increased cellular proliferation and differentiation, followed by remodelling of tissues, organ enlargement (organomegaly), and disturbances to metabolism. Most acromegaly patients experience a long delay between the appearance of the first symptoms of the disease, its diagnosis, and the start of the treatment (2). In consequence, there is a chronic exposure to increased GH and IGF-I levels leading to the development of typical acromegaly symptoms, and multiple systemic complications as cardiovascular, metabolic, respiratory, endocrine, and bone disorders (3, 4). Cardiovascular disorders leading to myocardial infarction and stroke, respiratory diseases, and cancers are the main reasons for premature mortality in this group (5, 6). Early diagnosis of acromegaly and its treatment is mandatory to avoid comorbid diseases and further complications leading to premature death. Radical surgery as well as pharmacological control of disease activity decrease mortality to that observed in the normal population (7, 8). Moreover, a lot of acromegaly patients suffer from decreased life quality despite cured or well-controlled disease activity (9, 10). Complications like diabetes, cardiovascular disease and vertebral fractures contribute to a significant reduction of patients’ quality of life (11, 12).

First guidelines about diagnosis and treatment of acromegaly complications were published in 2003 (3)⁠. Since then some updates emerged, with the most relevant in 2013 (13) and 2020 (14), recommending a change in approach to the disease supporting holistic view on an acromegaly patient with involvement of different types of specialist. As stated in screening for comorbidities should be performed at the time of diagnosis and repeated regularly.

The aim of our study was to perform an in-depth analysis of the gathered database of hospitalization in terms of acromegaly complication frequencies and their co-occurrence. Also, we investigated the changes in procedures used in diagnostics and treatment of acromegaly patients over an eighteen-year period, from 2000 to 2018.

## Materials and Methods

In this retrospective study, we analyzed data of 179 patients hospitalized in the Department of Endocrinology, Diabetes and Isotope Therapy in Wroclaw Medical University (Poland) in 1976 to 2018. The inclusion criteria were diagnosis of acromegaly according to Endocrine Society Guidelines (15) – elevated IGF-I levels and unsuppressed GH in OGTT at present or in the past. The study included patients with an established acromegaly diagnosis. The database of the patients was created by a single researcher and the registry included 560 records. Data came from patients’ hospitalizations at the Department of Endocrinology, Diabetes and Isotope Therapy in Wroclaw and the Neurosurgery Department in Warsaw. Registry included patients’ demographic characteristics, laboratory test results and performed procedures, as well as recommendations for the patients. The number of hospitalizations for each patient was between 1 and 16, with a mean of 3.136 for women and 3.151 for men. The average time of monitoring was 5 years.

Statistical analysis was performed using R for Windows, version 3.5.3.

### Occurrence of Micro- and Macroadenomas

For 132 patients adenoma sizes were available. An adenoma was classified as a macroadenoma if at least one of its sizes was greater than 10 mm. The comparison of micro- to macroadenomas between males and females was performed using the Chi-squared test.

### Comparison of Patients Who Underwent One *vs.* Several Surgeries

All comparisons were performed separately for male and female patients. The IGF-I concentrations and GH concentrations were compared using the Wilcoxon Rank Sum test. GH concentrations were log-transformed prior to analysis. The comparison was also performed with Student’s t-test (data not shown) - with consistent results. The comparison of complications occurrence (pituitary insufficiency, secondary hypogonadism, panhypopituitarism) between the groups was performed using the Chi-squared test.

### Comparison of Complication Frequencies Between Male and Female Patients

The comparison for each complication was performed using the Chi-squared test with Benjamin-Hochberg False Discovery Rate correction (BH).

### Analyzing Complications Occurrence With Respect to Patient Age

The analysis was performed separately for male and female patients. For each patient, we have found at what age the analyzed complication first occurred. Age values were categorized into 10-year ranges starting (0, 30], (30, 40] and so on till (80, 100]. The bins were then compared using Chi-squared with BH correction to see if the occurrence is related to patient age.

### Clustering Analysis of Complication’s Co-Occurrences

The heatmap was generated by performing agglomerative clustering first, to group patients that suffered from similar complications (this provided the patient clustering dendrogram), second, to group co-occurring complications (this provided the complications clustering dendrogram). In both cases, the Ward’s clustering algorithm was used (16) with binary distance metric.

P-value less than 0.05 was considered statistically significant. The study was approved by the local ethical committee.

## Results

**Figure 1:**
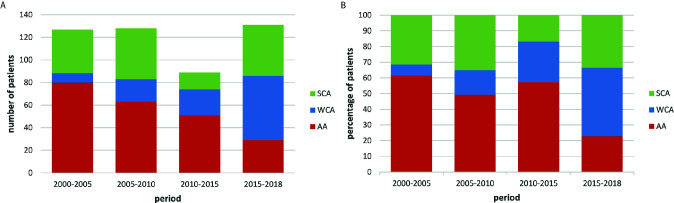
Distribution of patients with active, controlled and cured acromegaly over 5-year time frames. **(A)** – the numerical value; **(B)** – the percentage value AA, active acromegaly; WCA, well-controlled acromegaly; SCA, surgery cured acromegaly.

Among the group, there were 119 women (66%) and 60 men (34%). The median age of acromegaly diagnosis was 50.5 years old for women (age range, 20–78) and 46 for men (range, 24–76). During the last registered hospitalization, among patients with known disease status, 69 patients (39.7%) had cured acromegaly, 52 (29.9%) had still active disease and 53 (30.5%) were pharmacologically well-controlled. Over the years the proportion between active acromegaly patients and well-controlled patients decreased (**Figure 1**). In 5-year periods, analyzed from 2000 to 2018, there is a statistically significant decreasing trend in the number of active disease patients (80 vs. 29, 2000 to 2005 vs. after 2015, respectively; p=0.01), this is accompanied by an increasing fraction of well-controlled acromegaly patients - the trend of percentage of WCA cases was significantly increasing (7.1% vs. 43.5%, p=0.02).

In 132 patients, there was information about tumor size. Among patients with active acromegaly (102 patients), there was the predominance of macroadenomas (n=78, 76.5%). In this group, we found a higher ratio of incidence of macroadenomas to microadenomas in men (31/7) than in women (47/17), however, this difference was not statistically significant (p=0.49). In the tumor sizes analysis, we have found statistically significant correlations with IGF-I (p<0.000001) and GH levels (p<0.000001) (GH levels were log-transformed prior analysis - for details see Methods section) in the AA group.

139 patients underwent surgery: 110 were operated on once, 20 twice, 6 patients had three, and 3 had four surgeries. 134 patients had transsphenoidal and 10 transcranial surgeries. In 5 cases both types of operation were performed. 108 patients were operated once transsphenoidal, at 26 cases reoperations by the same method were performed.

**Figure 2:**
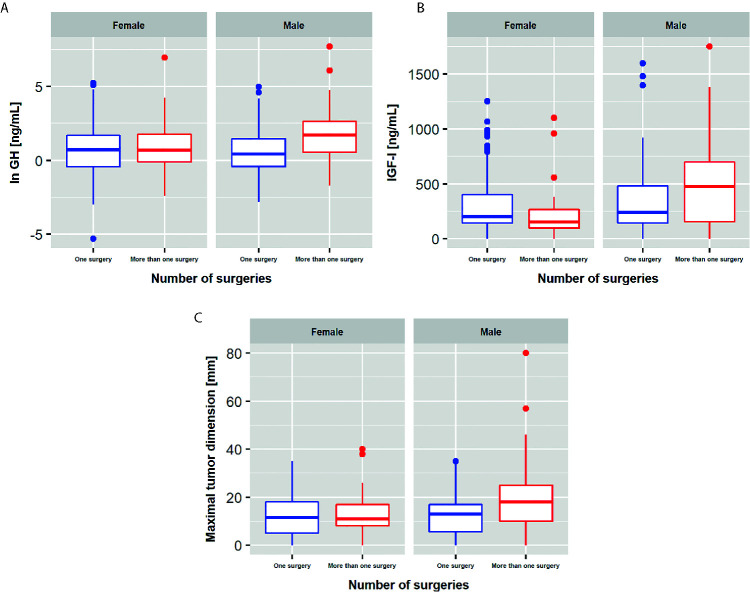
Differences between patients operated once and patients with reoperations. **(A)** GH concentrations; **(B)** IGF-I concentrations; **(C)** maximal tumor dimensions.

GH concentrations and tumor sizes (maximal tumor dimensions) were significantly higher among males with more than one surgery compared to operated only once (p=0.00006; p=0.04, respectively). This difference was not observed among females. Interestingly, IGF-I concentrations were significantly lower among females, but not among males, operated more than once in comparison to females with one surgery (p=0.01). These results are presented in **Figure 2**.

In most cases (98/179 – 54.7%) combination therapy was performed. The most often chosen option of treatment was a combination of surgical and pharmacological therapy (in 45.3%, 81/179). Combination of surgery with radiotherapy was performed in 2.2% (4/179) and pharmacotherapy with radiotherapy in 1.1% (2/179). In 6.1% (11/179) combination of all the three methods were applied. In some cases, only one method of treatment was applied – surgery in 24.6% (44/179) and pharmacotherapy in 13.4% (24/179).

**Figure 3:**
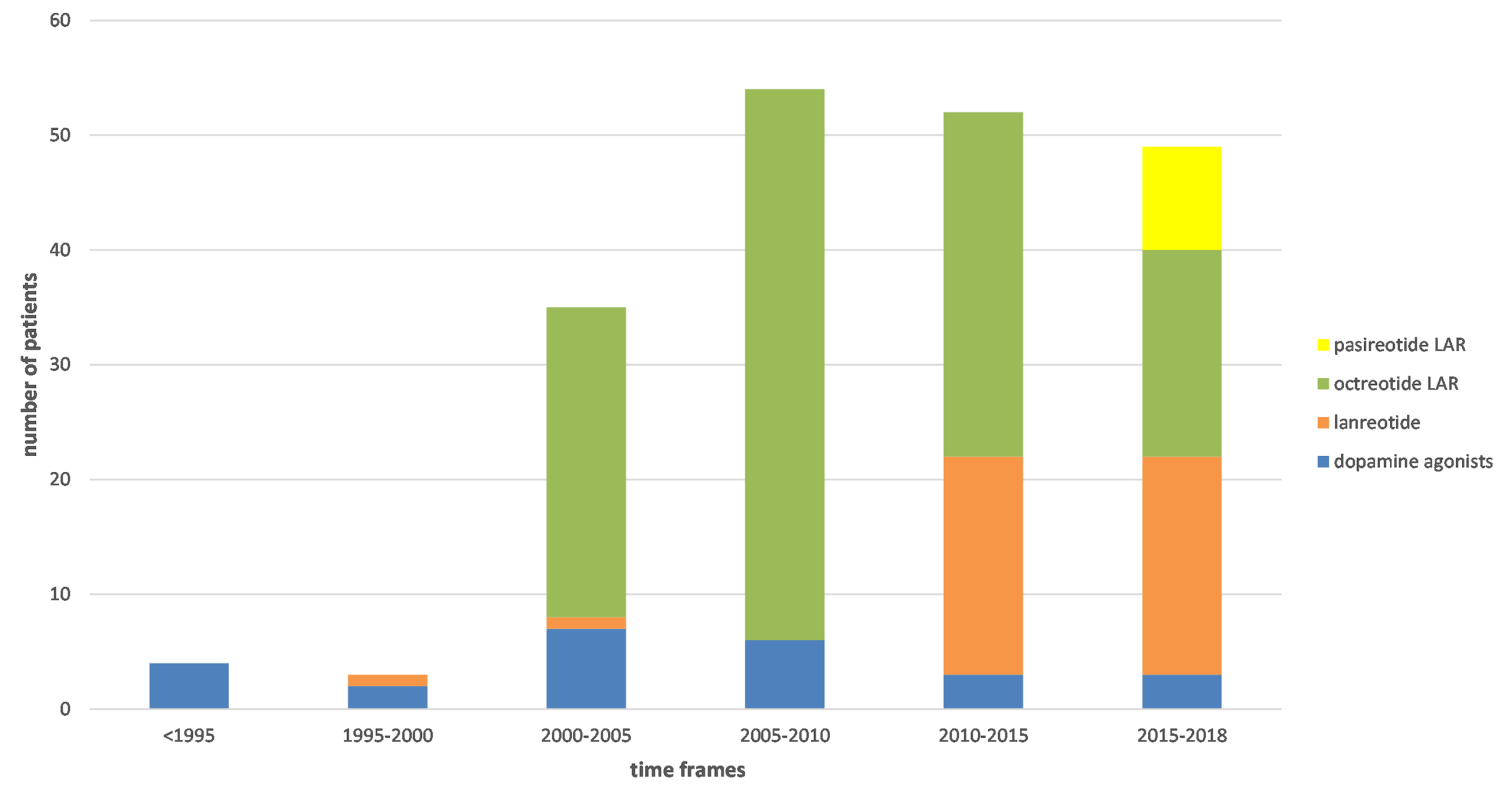
Type of used pharmacotherapy over 5-year time frames.

The type of pharmacological therapy used in patients underwent changes throughout the years. Sparsely available information regarding therapy used before 2000 indicates that most of the patients were treated with dopamine agonists (**Figure 3**). After 2000, somatostatin analogues became a dominant type of pharmacotherapy. Among them, up to 2015 octreotide LAR was preferred, and after 2015 – lanreotide Autogel became the most often chosen. Pasireotide LAR is used in few patients in recent years while pegvisomant was introduced in Poland later, in 2019.

### Complications of Acromegaly

Metabolic disorders (hyperlipidemia, diabetes, and prediabetes) were the most frequently diagnosed complications in our study, followed by cardiovascular diseases and endocrine disorders (goiter, pituitary insufficiency, osteoporosis). More detailed data about the frequency of occurrence of specific complications and their distribution in males and females are presented in **Table 1**.

**Table 1:** Prevalence of complications and their distribution in genders.

Complication	Number of patients	%	F/M	%F	%M
Lipid disorders	133	74	90/43	76	72
Hypertension	103	58	72/31	61	52
Goiter	93	52	65/28	55	47
Joint degeneration	72	40	46/26	39	43
Hypopituitarism	66	37	36/30	30	50
Secondary hypogonadism	33	18	13/20	11	33
Secondary adrenal insufficiency	46	26	25/21	21	35
Secondary hypothyroidism	40	22	21/19	18	32
Panhypopituitarism	18	10	6/12	5	20
Changes in echocardiograms*	61	34	36/25	30	42
Prediabetes**	61	34	33/28	28	47
Diabetes***	59	33	44/15	37	25
Cholelithiasis	51	28	36/15	30	25
Arrhythmias	35	20	20/15	17	25
Osteoporosis	22	12	15/7	13	12
Nephrolithiasis	22	12	13/9	11	15
Colonic polyps	21	12	13/8	11	13
Ischemic heart disease	12	7	11/1	9	2
Heart failure	11	6	6/5	5	8
Carpal tunnel syndrome	8	4	7/1	6	2
Sleep apnea	3	2	1/2	1	3

*Left ventricular and interventricular septum hypertrophy, diastolic dysfunction, valvular defects – mainly mitral valve regurgitation, improper atrial and ventricular dimensions.

**Diagnostic criteria were: fasting plasma glucose between 100 and 125 mg/dL or two-hour plasma glucose value between 140 and 199 mg/dL.

***Diagnostic criteria were: fasting plasma glucose ≥126 mg/dL repeated twice or two-hour plasma glucose value of ≥200 mg/dL during a 75-g oral glucose test (OGTT), or symptomatic hyperglycemia (weight loss, polyuria, polydipsia) and blood glucose ≥200 mg/dL.

Hyperlipidemia was the most frequent comorbid disease and metabolic disorder (74%). We analyzed the lipid profiles from the first hospitalizations of the patients with hyperlipidemia. In 126/133 cases we had complete data about cholesterol and triglycerides levels. 56 patients (44%) had high cholesterol (>200 mg/dL) with normal triglycerides levels (≤150 mg/dL). 20 patients (16%) had isolated hypertriglyceridemia, whereas the coincidence of hypercholesterolemia and hypertriglyceridemia was observed in 40 cases (32%). In 19 patients coexistence of hypertriglyceridemia with low levels of HDL (< 40 mg/dL in men and <45 mg/dL in women) was observed, however in 44 cases there is a lack of information about HDL results. Among cardiovascular diseases, hypertension occurred the most often (58%), and among endocrine – goiter (52%). Hyperlipidemia (90/119), hypertension (72/119), and goiter (65/119) were the most frequent complications in female patients. All the three occurred in more than half of the subgroup. In the male patients hyperlipidemia also dominated (43/60), hypertension was at the second place (31/60), but at third, there was hypopituitarism (30/60). Similarly, all of the three were observed in at least half of the subgroup.

Pituitary insufficiency (insufficiency of one pituitary axis), secondary hypogonadism, panhypopituitarism, and prediabetes occurred more frequently in males than females (p=0.01; p<0.0005; p=0.003; p=0.01, respectively). The analysis showed that in males pituitary insufficiency, as well as secondary hypogonadism was more common in patients after radiotherapy (p=0.04; p=0.008, respectively). Radiotherapy did not have a significant effect on the incidence of panhypopituitarism (p=0.09). The size of tumor did not have any impact on the incidence of pituitary insufficiency, secondary hypogonadism, panhypopituitarism (p=0.23; p=0.52; p=0.21, respectively). Among males and females with more than one surgery, pituitary insufficiency, secondary hypogonadism, and panhypopituitarism were observed more frequently compared to males and females with one operation (for males p=0.001; p=0.002; p=0.009, respectively, for females p=0.0001; p=0.04; p=0.005, respectively).

We further explored if specific complications occurred more frequently in specific age ranges within genders. In females, there was a higher occurrence of hypertension, diabetes, and osteoporosis in a range of 60–70 years. In males, hypertension was diagnosed a decade earlier than in females (in range 50–60). Additionally, we analyzed the prevalence of each complication according to a distribution of the acromegaly activity during the last hospitalization (**Table 2**). In order to analyze the coincidence of complications at a more general level, we aggregated complications into the three groups: metabolic, cardiovascular and endocrine (**Table 3**). The coincidence of the groups of complications revealed that 50.8% of patients suffered from at least one complication from each group. Frequency of co-occurrence of metabolic and cardiovascular disorders as well as metabolic and endocrine diseases was 15.5% for both. Only 5.0% of patients did not suffer from any disease belonging to those main groups.

**Table 2:** Prevalence of complications and distribution of the disease activity during the last hospitalization.

Complication	AA (%)	WCA (%)	SCA (%)	Total
Lipid disorders	32 (31)	29 (28)	43 (41)	104
Hypertension	29 (30)	34 (35)	34 (35)	97
Goiter	20 (24)	36 (43)	28 (33)	84
Joint degeneration	20 (33)	24 (39)	17 (28)	61
Hypopituitarism	19 (33)	19 (33)	20 (34)	58
Secondary hypogonadism	9 (33)	8 (30)	10 (37)	27
Secondary adrenal insufficiency	11 (29)	13 (34)	14 (37)	38
Secondary hypothyroidism	12 (32)	12 (32)	13 (35)	37
Panhypopituitarism	5 (33)	3 (20)	7 (47)	15
Changes in echocardiograms	8 (23)	14 (40)	13 (37)	35
Prediabetes	15 (54)	8 (29)	5 (18)	28
Diabetes	18 (34)	27 (51)	8 (15)	53
Cholelithiasis	11 (24)	25 (54)	10 (22)	46
Arrhythmias	10 (50)	7 (35)	3 (15)	20
Osteoporosis	6 (32)	6 (32)	7 (37)	19
Nephrolithiasis	6 (38)	3 (19)	7 (44)	16
Colonic polyps	1 (5)	10 (53)	8 (42)	19
Ischemic heart disease	2 (25)	5 (63)	1 (13)	8
Heart failure	3 (38)	3 (38)	2 (25)	8
Carpal tunnel syndrome	0 (0)	4 (67)	2 (33)	6
Sleep apnea	0 (0)	2 (67)	1 (33)	3

AA, active acromegaly; WCA, well-controlled acromegaly; SCA, surgery cured acromegaly.

**Table 3:** Coincidence of complications belonging to the metabolic, cardiovascular and endocrine categories.

Metabolic	Cardiovascular	Endocrine	N	%
1	1	1	91	50.8
1	1	0	28	15.6
1	0	1	28	15.6
1	0	0	11	6.1
0	1	1	4	2.2
0	0	1	8	4.5
0	0	0	9	5.0

**Figure 4:**
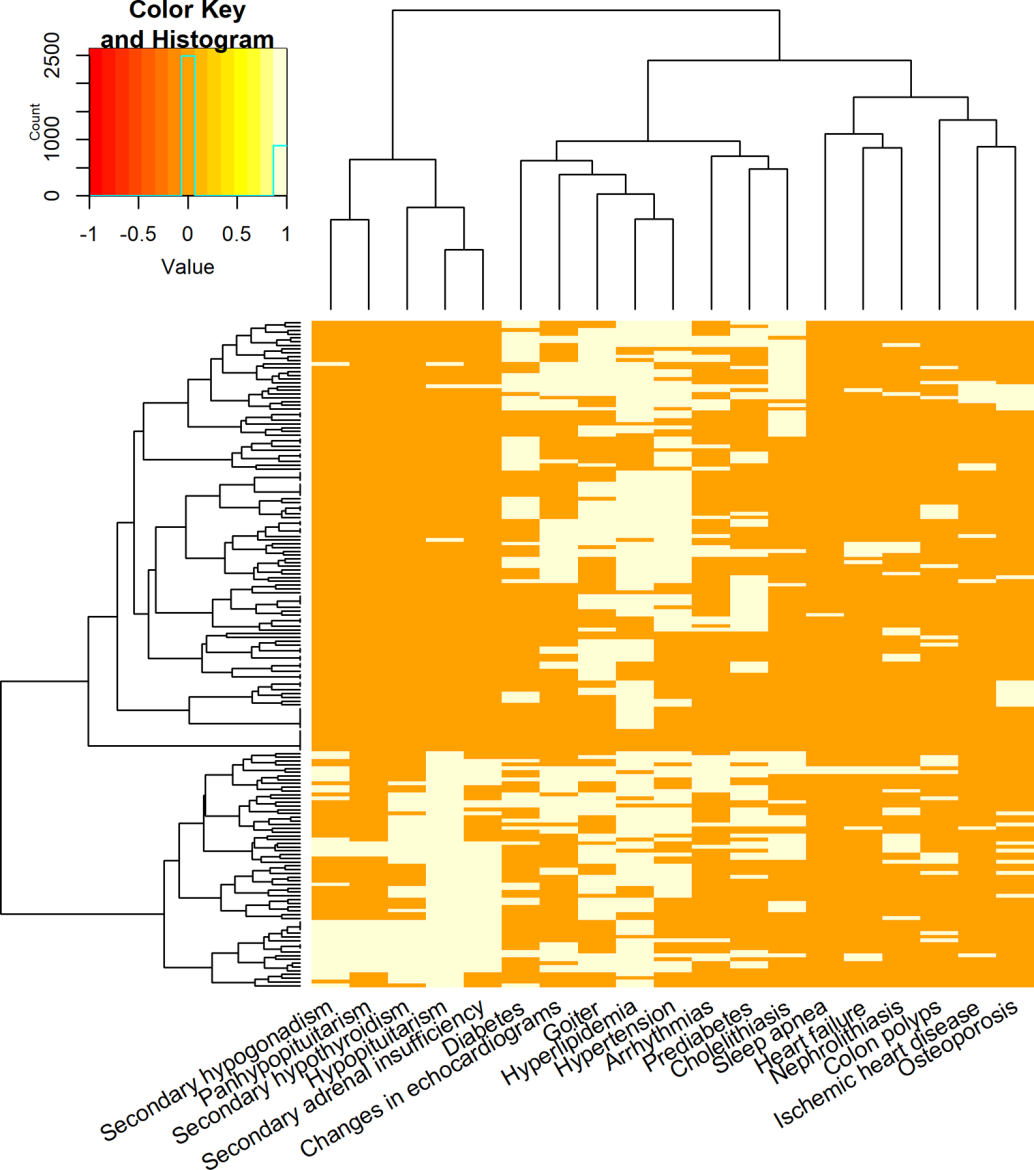
Heatmap of particular complications co-occurrence. Each row in the heatmap denotes a patient. Each column is a complication. Bright fields mark complications that a patient suffered from. In addition, the dendrograms on the left-hand side and the top panel present grouping of patients, and grouping of complications, respectively.

Furthermore, an evaluation of the coexistence of particular complications by using heatmap clustering was performed (**Figure 4**). It revealed that types of pituitary insufficiency co-occurred more frequently. On this map, also a tendency to the coexistence of diabetes, hyperlipidemia, hypertension, and heart remodelling was observed. In addition, a cluster including prediabetes and cholelithiasis was obtained. Groups of diseases distinguished from the clustering are presented in **Table 4**.

**Table 4:** Groups of complications obtained from heatmap clustering.

I	hypopituitarism, secondary hypogonadism, secondary hypothyroidism, secondary adrenal insufficiency, panhypopituitarism
II	diabetes, hyperlipidemia, hypertension, changes in echocardiograms, goiter
III	prediabetes, arrhythmias, cholelithiasis
IV	osteoporosis, ischemic heart disease, heart failure, sleep apnea, colonic polyps, nephrolithiasis

### Analysis of Diagnostic Procedures

We investigated the frequencies of diagnostic procedures performed or ordered during hospitalizations in the Endocrinology Department for diagnostics and monitoring purposes. They are listed in **Table 5**. The results of colonoscopies are presented in **Table 6**.

**Table 5:** Number of procedures performed during hospitalizations in the Endocrinology Department.

Type of procedure	Number of procedures/496 hospitalizations	%
BP measurement	481	97.0
Lipid profile	462	93.1
Fasting glucose	453	91.3
OGTT (if no diabetes)	299	79.5
Electrocardiogram	391	78.8
Densitometry	235	47.4
Hormonal profile*	213	42.9
Abdomen ultrasound	224	45.2
Thyroid ultrasound	198	39.5
Echocardiogram	111	22.4
Colonoscopy	43	8.7

*FSH, LH, PRL, TSH, fT4, morning cortisol, testosterone (in men), estradiol (in women).

**Table 6:** The findings of colonoscopy in analyzed acromegaly patients.

Results	Number of patients
Normal endoscopic appearance	7
Colonic polyps	19
Proximal colon to splenic flexure	5
Distal colon to splenic flexure	9
Proximal and distal colon	3
Rectum	2
Diverticulosis of the sigmoid colon	5
Grades Internal hemorrhoids	14
I	6
II	5
III	1
IV	2
External hemorrhoids	2
Colitis	5
Ulcerative colitis	1
Spastic colon	1
Irritable bowel syndrome (IBS) suspicion	2

I, II, III, IV: grades of internal hemorrhoids.

We analyzed the changes in numbers of procedures in 5-years periods. We have found that there was an increasing trend in the number of performed thyroid ultrasounds (p=0.01). During this period, the number of performed abdomen ultrasounds correlated positively with the detectability of cholelithiasis (p=0.02). There were no significant trends in other procedures.

## Discussion

Acromegaly complications are the main factors contributing to lifespan and its quality in patients, so active diagnostics and treatment are essential. The knowledge about comorbidities of acromegaly has changed in the past decades. For this moment, radical surgical treatment or even pharmacological control of the disease can enable reversibility of some complications, but only if those are recognized and treated in early stages. So, it is very important to shorten the delay in the diagnosis of acromegaly. In addition, it is essential to improve the regularity of diagnostic exams that help us to recognize comorbidities and treat them earlier. Obtaining a strict control of hormone excess is the best strategy to limit the development of complications of acromegaly.

### Patients Characteristics

In the population of our patients, we can observe substantial domination of females. Similar outcomes were reported in Polish multicenter study (17), as well as in other European registries (18–23). Not statistically significant, but also a higher ratio of incidence of macroadenomas to microadenomas in men compared to women was detected in the French population (21).

As we expected, positive correlations between maximal tumor dimensions and IGF-I and GH concentrations were obtained in patients with an active phase of the disease. Similarly, such correlations were observed for acromegaly patients before surgery (24). Adenoma measurements correlated positively with GH levels, but not with IGF-I levels in Tirosh et al. (25). and Evran et al. (26). studies, while Schwyzer et al. obtained contrary results with a significant correlation between preoperative tumor volume and IGF-I, but not with GH level (27). In Tirosh et al. study (25) an association between tumor volume and hypogonadism was determined, more pronounced in males, which was not obtained in our study. In that study also an association between size of tumor > 10 mm before the operation and the need for re-operation was observed. In our research, we also observed dependence between the maximal tumor diameter and reoperations in males, but in that analysis, we used data of maximal tumor diameter from all MRI results, also after an operation, not only before.

### Acromegaly Treatment

Over the years, we observed a reduction in the number of patients with active acromegaly and an increase of pharmacologically controlled individuals. Consistent outcomes were depicted in the French registry (21). Many of the patients required additional treatment due to non-radical operation or recurrence. Sometimes decisions about reoperation are afflicted by difficult localization of the tumor, risk of damage to important structures (e.g. optic chiasm) or patients’ coexisting diseases. Introduction and wide availability of somatostatin analogues allowed to achieve good control of disease activity and reduction in the occurrence of serious complications.

### Epidemiology and Pathogenesis of the Main Groups’ Complications

#### Cardiovascular Complications

Cardiovascular complications are the main causes of mortality in acromegaly patients (2, 3, 8, 13). Cure or even control of disease activity decrease the frequency and severity of comorbidities from this group. Some of them can even be totally reversed (28)⁠. In our study hypertension was the dominant complication from the cardiovascular group – it occurred in 58% of patients, which is consistent with previous studies. It has been reported that it occurs approximately in one-third of patients, ranging from 18 to 60% (4, 17, 28–30). What is more, the prevalence that we obtained is higher than in the general Polish population, which is 42.7% (31). The etiology of hypertension in acromegaly is multifactorial. It includes, inter alia: increased plasma volume, hypertrophy of vascular smooth muscles, vascular stiffness, and endothelial dysfunction (28, 29)⁠. Management of hypertension in acromegaly patients does not differ from that applied in the general population (14).

In other databases, left ventricle hypertrophy has been observed in 70–90% of reported cases [but only in 15% of patients in Liege Acromegaly Survey Database (19)] while valve diseases in 20 to 75% (4, 28). In our study changes in echocardiograms (left ventricular and interventricular septum hypertrophy, diastolic dysfunction, valvular defects – mainly mitral valve regurgitation, improper atrial and ventricular dimensions) were found in 34% of the cases. The excess of GH and IGF-I is known to contribute to myocardial concentric hypertrophy, which leads to a decrease in the cardiac output and heart failure (29). We observed heart failure only in 6% of the cases, whereas the prevalence of this complication was previously described in approximately 10% of the cases (4). These differences may be a consequence of performing fewer examinations, including echocardiograms in the past.

There are conflicting data regarding acromegaly’s contribution to a higher occurrence of ischemic heart disease (32). The probable origin of this complication is not associated with GH and IGF-I excess levels, but with other comorbidities like dyslipidemia, insulin resistance, hypertension, and sleep apnea (28). Ischemic heart disease was diagnosed in 7% of our patients. The incidence in previous registries was between 2.5 and 12% (17, 19, 28).

The most common arrhythmias observed in acromegaly are atrial fibrillation, sinus or ventricular tachycardia, sick sinus syndrome, and ventricular extrasystoles (28). Arrhythmias were reported to affect 7% to 48% of patients (4, 28). In our study, we observed them in 20% of the cases. The number of performed electrocardiograms was high (78.8%), but it is a short time examination and it is possible that some disturbances might be detected only in Holter ECG.

#### Metabolic Complications

The prevalence of diabetes in acromegaly patients is 12% to 53% (17, 19, 30, 33, 34). We observed diabetes in 33% of the cases and prediabetes in 34%, which is much higher than in the general Polish population (6.97%) (25). In Warncke’s study, it has been reported that in acromegaly diabetes affects younger patients (50.1 years) than in the general population (59 years) (35). Comparable results were observed for the Italian population (20). In our research, the dominant age of diabetes diagnosis was 60 to 70 and 50 to 60 years for prediabetes, respectively. Factors that contribute to disturbances in glucose metabolism in acromegaly are complex (33). GH is an antagonist hormone to insulin, which increases lipolysis and gluconeogenesis and predisposes to insulin resistance, which is the main factor in diabetes origin (33, 36). Normalization of GH concentration decreases glycemia and improves insulin sensitivity (37). It is important to highlight that treatment with somatostatin analogues, especially pasireotide, may impede control of diabetes and increase insulin resistance (13, 38). Diabetes therapy in acromegaly does not differ from generally used in type 2 diabetes (2, 19)⁠⁠⁠.

Dyslipidemia, depending on the source, affects 13-51% or even up to 71% of acromegaly patients (8, 35). GH induces lipolysis and increases levels of plasma free fatty acids (8). Hypertriglyceridemia and decreased HDL level are the main lipid abnormalities in acromegaly (8, 36). In our study dyslipidemia was the most frequent complication in the whole group and it was observed in 74% cases, so it was even higher incidence compared to the previous studies (8, 35). Moreover, we observed elevated triglycerides in 48% cases, which is also higher in comparison to the other reports (4).

#### Endocrine Complications

There is a proven correlation between thyroid gland volume and GH and IGF-I levels (39, 40). The prevalence of goiter in acromegaly patients reaches between 25 and 92% of the cases (17, 19, 39, 41, 42). We observed this complication in 52% of our patients. Furthermore, a predisposition for the autonomous function of some nodules in nodular goiter contributes to hyperthyroidism (39)⁠. Some studies suggest a higher incidence of thyroid cancer in acromegaly (39, 43). In the presented series, we diagnosed one follicular thyroid cancer and one papillary thyroid cancer.

Hypopituitarism is caused by compression or damage of the pituitary gland by an expansion of adenoma or it is an iatrogenic complication that appears after surgery or radiotherapy. The prevalence of hypopituitarism in our study was 37% and it dominated in the male population. Similar prevalence of hormonal deficiency was reported in the Belgian registry (44). On the other hand, for example in the USA database, this complication was observed only in 16.6% of the cases (30). Secondary adrenal insufficiency was the most frequent alternation in our research. What is more, we observed hypopituitarism in 64.7% of patients after radiotherapy. It is known that radiotherapy increases the risk of hypopituitarism – in this group it is diagnosed in more than half of the patients (36, 44). In contrary to the Italian registry (20), we observed the significant impact of radiotherapy only for hypogonadism in males. Hypopituitarism, secondary hypogonadism and panhypopituitarism were revealed more often in the male population. In the Italian registry, hypogonadism also predominated in males (20).

Acromegaly is also associated with secondary osteoporosis and fractures. Our research revealed osteoporosis in 12% of the cases. We did not have complete information about fractures in the analyzed group of patients. In previous studies, osteoporosis was observed in 12-32% of patients (19, 45). It is known that a higher risk of fractures may occur despite normal bone mineral density (BMD) (46). So, we still need new tools to estimate the risk of fractures in acromegaly. Assessment of bone quality can improve this evaluation. Recently introduced trabecular bone score (TBS) method is a promising tool, which enables to assess bone tissue microarchitecture (47, 48).

#### Coexistence of Complications

In more than half of patients, we observed co-occurrence of cardiovascular, metabolic and endocrine complications. This finding illustrates that they are common problems in acromegaly and acromegaly patients often require complex healthcare and multi-component therapy. Cardiovascular and metabolic, as well as metabolic and endocrine complications co-occurred more frequent than cardiovascular and endocrine disorders. The coincidence of hypertension, heart structural changes, hyperlipidemia and diabetes was also exemplified in heatmap clustering evaluation. Besides the impact of GH and IGF-I excess on cardiovascular and metabolic diseases, similar environmental factors contribute to the origin of morbidities classified in those groups. We speculate that obtained cluster of prediabetes and cholelithiasis coexistence may indicate on the group of patients treated with somatostatin analogues. Moreover, in the analysis of disease activity (**Table 2**), diabetes and cholelithiasis were observed in more than 50% in the well-controlled acromegaly patients treated with somatostatin analogues.

#### Diagnostics of Complications

We decided to determine if patients from our department underwent procedures that are recommended in the guidelines. According to current worldwide and Polish guidelines (14, 49) BP measurement, fasting glucose or OGTT should be performed every six months; ECG, echocardiography, Epworth scale, spine X-ray, lipid and hormonal profile (towards hypopituitarism diagnostics) and Acromegaly Quality of Life Questionnaire (AcroQoL) annually; thyroid ultrasound every 1 to 2 years; DXA every 2 years and colonoscopy every 10 years. In order to do that we calculated the general number of each procedure during 496 hospitalizations (**Table 5**). According to obtained results BP measurement, ECG, lipid profile, fasting glucose, or OGTT were performed the most often. The least frequent examinations were colonoscopy and echocardiogram. None of our patients had polysomnography. This might be a result of the clinic profile. Polysomnography, colonoscopy and echocardiogram require the presence of other specialists to be performed. Unrecognized sleep apnea, colon cancer or heart failure can lead to serious consequences. It is important to highlight that in care of acromegaly patients, the involvement of different types of specialists is needed and that each patient should obtain referrals for additional consultations when needed. In 21 patients (12%) (in 19 cases colonoscopy was recommended in the Endocrinology Department, in 2 an information about diagnosis was included in the medical histories) colonic polyps were observed. Colonic polyps are the most frequent types of tumors in acromegaly (39), so obtained rate indicates that this diagnosis could be underestimated in the group of our patients, but it is similar to observed in the Liege Acromegaly Survey Database (19). Hemorrhoids and diverticulosis, which also are reported to be associated with GH and IGF-I excess (4), were detected in 37 and 12% of examinations, respectively. The number of performed abdomen ultrasounds correlated positively with cholelithiasis detectability. Cholelithiasis might be a side effect of somatostatin analogues treatment, thus ultrasound is recommended, especially in pharmacologically treated group. In addition, the medical histories lack the information regarding the performance of AcroQoL, thus we speculate that it has not been conducted. Nowadays recommendations and studies emphasize that life quality assessment is very important in the evaluation of the effectiveness of treatment in acromegaly (10, 11, 14).

## Conclusions

In our population we observed female predominance. Over the years we noticed a reduction in the number of patients with active acromegaly and an increase of patients with pharmacologically controlled disease. More than 50% of patients demonstrated a coexistence of cardiac, metabolic and endocrine disturbances and what is important to decline only 5% of patients did not suffer from any disease from those main groups. For these reasons, an obtain a strict control of hormone excess is the best strategy to limit the development of complications of acromegaly.

## Data Availability Statement

The original contributions presented in the study are included in the article, further inquiries can be directed to the corresponding author.

## Ethics Statement

The studies involving human participants were reviewed and approved by Bioethics Committee of the Wroclaw Medical University, Wrocław, Poland. Written informed consent for participation was not required for this study in accordance with the national legislation and the institutional requirements.

## Author Contributions

MR, AJ-P, and MB contributed to the study conception and design. Material preparation and data collection were performed by MR, GZ, AJ-P, JH-Ż, MK, and IB. MR created the database. BK performed the statistical analysis. MR, AJ-P, JH-Ż, and BK interpreted the results. The first draft of the manuscript was written by MR and AJ-P and corrected by JH-Ż, BK, GZ, and MB. All authors contributed to the article and approved the submitted version.

## Funding

This study was supported by Statutory Activities by the Minister of Science and Higher Education (grant number SUB.C120.21.025).

## Conflict of Interest

The authors declare that the research was conducted in the absence of any commercial or financial relationships that could be construed as a potential conflict of interest.
